# Investigating the method of selection of background pixel values for the calibration of EBT‐XD film dosimetry

**DOI:** 10.1002/pro6.1236

**Published:** 2024-08-11

**Authors:** Sathiya Raj, Nithya Shree, Ganesh Kadirampatti

**Affiliations:** ^1^ Radiation Physics Kidwai Memorial Institute of Oncology Bangalore India

**Keywords:** Film dosimetry, EBT‐XD film, Calibration curve

## Abstract

**Purpose:**

This study investigates three different calibration methods for the selection of background pixel intensity.

**Methods:**

Film‐by‐Film (FBF) Method: Each film serves as its own control. Batch‐by‐Film (BBF) Method: A single film is used as a control for all calibration films. Generic (GEN) Method: A generic value (65535) is used as the background pixel value for all calibration films.Three calibration curves were established for the red, green, blue, and RGB channels, and the Radbard NIH (image) curve‐fitting model was used to predict the dose. Sensitivity at different dose levels was quantified by calculating the first derivative of each color channel.

**Results:**

The GEN method exhibited a difference of up to 6% between the predicted and delivered doses below 2 Gy. The changes in optical density when using the GEN method differed significantly (*p*<0.0001) from those of the FBF and BBF methods. In the dose range 5–30 Gy, the percentage difference between the predicted and delivered doses for the FBF, BBF, and GEN methods was within 2%. Both the red and green channels demonstrated higher sensitivity than the blue channel over the dose range of 2–30 Gy.

**Conclusions:**

The FBF method is more accurate than the BBF and GEN methods because it accounts for inter‐film variations. The Radbard NIH (image) curve‐fitting function proved suitable for predicting the dose for all the three calibration methods.

## Graphical abstract

Film Exposure: Film exposure was conducted immediately after linac reference dosimetry. Film irradiation was performed using the Elekta Infinity linac with a 6 MV x‐ray beam energy. SSD was set to 100 cm, and the field size was opened to 10 cm×10 cm. Films were placed at a 5 cm depth in the slab phantom. Since MUs were obtained from the same setup through the TPS, there was no need to keep the films at a water‐equivalent depth in the slab phantom. A Farmer‐type ion chamber was placed at the bottom of the phantom to monitor any unusual output fluctuations in the linac. The chamber was positioned well below the depth of the film to avoid any perturbation that might affect the film dose. The films were exposed to doses of 0 (control), 0.5, 2, 5, 8, 12, 16, 20, 25, and 30 Gy.

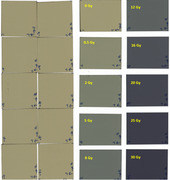

## 1 INTRODUCTION

External Beam Therapy (EBT) films have been established as crucial tools for various radiotherapy applications, both quantitatively and qualitatively. These applications include patient‐specific quality assurance (PSQA)^,^ ^1^, ^2^ multileaf collimator QA,^3^, ^4^ radiation isocenter verification,^5^, ^6^ small‐field output factor measurements,^7^, ^8^ and in vivo dosimetry.

EBT films have undergone continuous improvement, leading to the development of enhanced versions such as EBT2, EBT3, EBT4, and EBT‐XD films.^10^ Each iteration aims to overcome the limitations of its predecessor. In particular, EBT‐XD film was introduced for high‐dose radiotherapy applications, and has a useful dose range extending from 0.4 Gy to 40 Gy,^11^ whereas for EBT2, EBT3 and, EBT4 dose range is 0.2 Gy to 10 Gy.

Numerous researchers have extensively characterized EBT‐XD films and investigated aspects such as dose response, dose rate dependency, energy dependency, post‐exposure variations, and the effect of film orientation on the scanner.^12^, ^13^, ^14^, ^15^, ^16^

In general, the accuracy of EBT film dosimetry is influenced by a multitude of factors, including the film type, active components within the film calibration process, film scanning orientation, inter‐ and intra‐film variations, scanner uniformity, curvature of the film on the flatbed scanner, and the size of the region of interest.^10^ Owing to the interplay between these factors, there is an inherent degree of uncertainty associated with film dosimetry.

EBT‐XD films have shown improvements in addressing the lateral response artifact (LAR) effect.^17^ Furthermore, investigations into the effect of film orientation (portrait/landscape) on the dose response, particularly when scanning the film from one direction to another (flipping the film), have shown minimal differences.^14^

Calibration of the EBT‐XD film is of paramount importance, particularly in the selection of the net optical density (net OD) for unexposed films. However, literature on the selection of net OD values for EBT‐XD film calibration is limited.^15^, ^16^, ^17^, ^18^ This limitation prompted an exploration of how the selection process of net OD accounts for background signals influencing the response of the EBT‐XD film.

## 2 MATERIALS AND METHODS

### 2.1 Film cutting procedure

**FIGURE 1:**
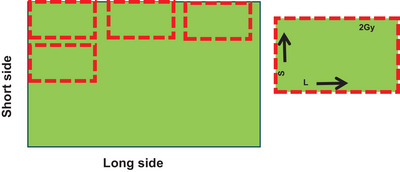
Full sheet of film (left); small piece of calibration film from the full sheet and with appropriate markings (right).

EBT‐XD films (Ashland Specialty Ingredients, Wilmington, DE, USA‐lot number:04052202) were used in this study. A paper cutter was used to cut the film. A full sheet of film (20.32 cm× 25.4 cm) was placed on the cutter and a small strip of film (approximately 4 cm× 5 cm) was cut into 10 pieces for calibration purposes. The sides of the films were labeled on each film strip, with “S” for the small side and “L” for the long side. Each film was numbered to identify the dose levels. Figure 1 shows the marking and cutting methods for the films used for calibration.

### 2.2 Linac calibration

Before irradiating the films, linac reference dosimetry was conducted using an IBA‐FC65‐G Farmer‐type ion chamber (0.65 cc) in a water phantom (30 cm× 30 cm× 30 cm). The source‐to‐surface distance (SSD) was set to 100 cm, and the field size was set to 10 cm × 10 cm. The chamber was placed at a calibration depth 10 cm below the water's surface. To minimize output fluctuations, the meter readings for each polarity were repeated six times by delivering 100 monitor units (MU), and all necessary correction factors were applied, as per International Atomic Energy Agency (IAEA) Technical Reports Series No. 398.^19^

### 2.3 MU calculation

A 40 (l) × 40 (b) × 15 (h) slab phantom was scanned using a Philips Big Bore CT scanner (Netherlands) with settings of 120 kVp, 200 mAs, and 3 mm slice thickness. The scanned phantom images were exported to Treatment Planning System (TPS) MONACO (Sweden) version 5.51.10. The MUs required to deliver the known doses were obtained from the TPS at dose levels of 0.5, 2, 5, 8, 12, 16, 20, 25, and 30 Gy for a 6MV photon beam. The calculation grid size was set to 0.25 cm. The dose was calculated at a depth of 5 cm from the surface of the phantom, and the SSD was set to 100 cm. The collapsed‐cone algorithm was used to calculate the MUs for each dose. The calculated dose was then verified using the same setup with a Farmer‐type ion chamber (0.65 cc). The dose variation was less than 0.2% compared to that of the TPS, which gave confidence in the output delivery of the linac on the day of film exposure.

### 2.4 Film exposure

**FIGURE 2:**
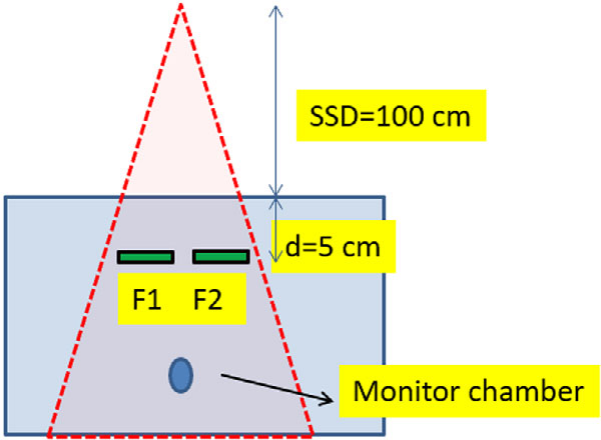
Film irradiation setup (F1 and F2; film 1 and film 2).

**FIGURE 3:**
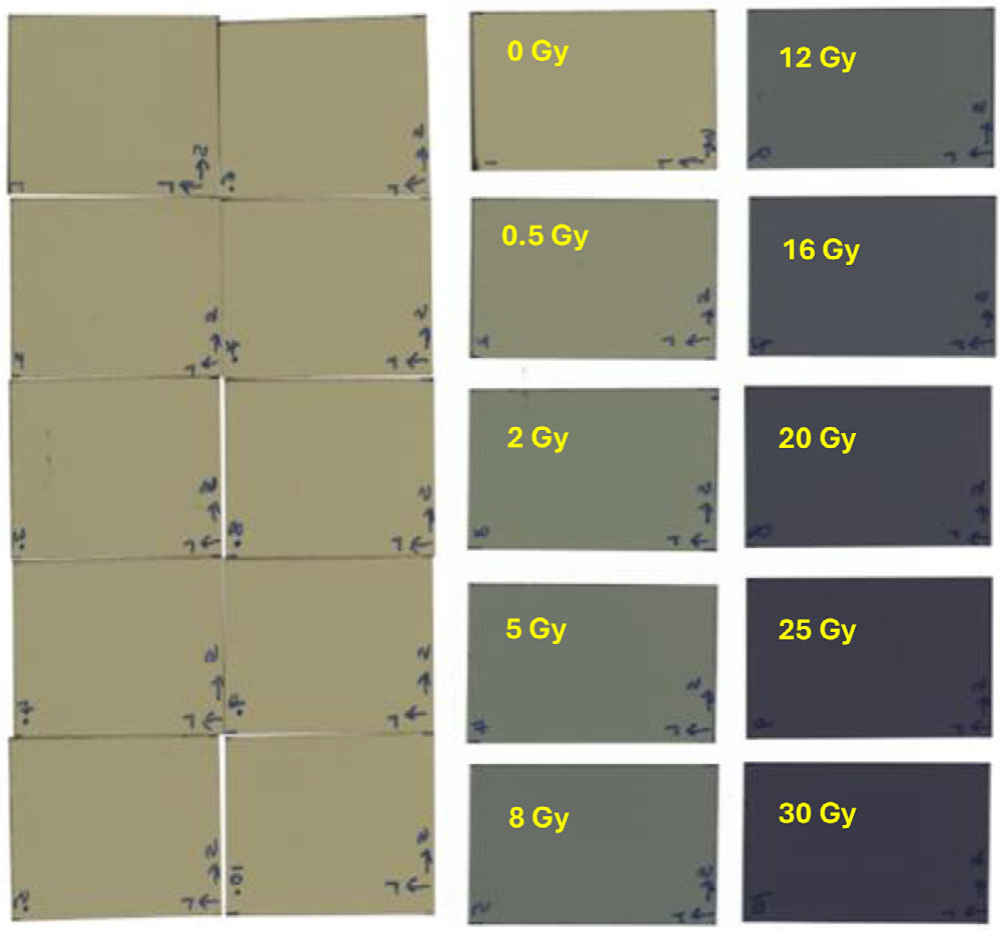
Film samples before exposure (left) and after exposure (right).

Film exposure was conducted immediately after the linac reference dosimetry. Film irradiation was performed using an Elekta Infinity Linac with a 6 MV X‐ray beam energy. The SSD was set to 100 cm, and the field size to 10 cm × 10 cm. The films were placed at a depth of 5 cm in the slab phantom. Because the MUs were obtained from the same setup using TPS, there was no need to keep the films at a water‐equivalent depth in the slab phantom. A Farmer‐type ion chamber was placed at the bottom of the phantom to monitor unusual output fluctuations in the linac (Figure 2). The chamber was positioned well below the depth of the film to avoid perturbations that could affect film dose. The films were exposed to doses of 0 (control), 0.5, 2, 5, 8, 12, 16, 20, 25, and 30 Gy. Figure 2 shows the experimental setup for film irradiation, and Figure 3 shows the films before and after exposure.

### 2.5 Film scanning protocol

The film scanner and associated scanning protocol can significantly affect the dosimetric results. Factors such as scanner uniformity across the scanning surface, noise levels, absence of a glass compression plate, scan resolution, lateral position effect, and warm‐up time play crucial roles. More detailed information about the scanning parameters that influence dosimetric results can be found in the American Association of Physicists in Medicine (AAPM) Task Group Report no. 235.^10^ Given that these parameters affect the scanner, we carefully handled the film and performed scans accordingly.

The exposed films were scanned using an EPSON Expression 12000XL flatbed scanner. All calibration films were scanned after a 24‐hour post‐irradiation period. Recognizing that EBT‐XD films tend to have some degree of curvature, which could result in less contact with the scanner, a 2 mm thick glass compression plate was placed over the films to ensure good contact with the scanner bed. The scanner was set to transmission mode with a 48‐bit TIFF image (each channel had 16 bits), and the scan resolution was set to 75 dots per inch. All films were positioned within 8 cm of the center of the scanner to avoid lateral scanner artifacts and color corrections were turned off. Initially, seven dummy scans were performed without a film as a warm‐up. Afterwards, the scan was performed seven times with a film, with the first two scans omitted, to extract pixel values (PVs) for each exposed and unexposed film.

### 2.6 Calibration curve generation

The films were imported into the ImageJ software to separate the color channels (Red, Green, and Blue). For each film and color channel, four regions of interest (ROIs) were selected to obtain mean PVs. The mean PVs of the four ROIs were considered as the final PV for a given dose. Three types of calibration curves were generated.

1. For film‐by‐film calibration (FBF), each calibration film was scanned before irradiation and its own PVs (I_unexp_‐before irradiation) were used as the background. The PVs were converted to net OD using Equation 1:
2. For batch‐by‐film calibration (BBF), a common film was used as the control for a batch of films, and the PVs of the control film served as the background for all calibration films. The dose and net OD relationships were established using Equation 1.
3. For the generic calibration method (GEN), a generic PV value (65535) was used as the background (I_unexp_) for all calibration films. Calibration curves were generated using this method, and the dose and net OD relationships are given by Equations 3 and 4.
(1) $$netOD\;=log_{10}\;\left(\frac{I_{unexp}}{I_{exp}}\right)$$

here, *I*_unexp_ and *I*_exp_ are the PVs of unexposed and exposed films, respectively.

The unexposed film of the 16‐bit image had gray‐scale values ranging from 0 to 2^16‐1^ (= 65,535) ^18^. Thus, 65,535 (*I*_unexp_) was used as the PV of the unexposed film.

(2) $$netOD\;=log_{10}\;\left(\frac{65,535}{I_{exp}}\right)$$

For the RGB channel calibration curve, the PVs were averaged over the red, green, and blue channels, and the following equation was used to obtain the net OD:

(3) $$netOD\;=log_{10}\;\left(\frac{I_{unexp\left(Avg:RGB\right)}}{I_{exp\left(Avg:RGB\right)}}\right)$$

(4) $$\;I_{unexp/exp\left(Avg:RGB\right)}=\frac{PV_{R}+PV_{G}+PV_{B}}{3}\;$$

### 2.7 Uncertainty analysis

Reference dosimetry was conducted prior to film exposure and uncertainties were assessed. Four pieces were cut from a single sheet of film, and the standard deviation of the mean PVs was calculated to estimate the inter‐film uncertainty. Three pieces of film were scanned five times, and the average PVs of the different ROIs were determined. To estimate scanner reproducibility, the standard deviation was calculated for the averaged PVs. The use of a compression plate eliminated the uncertainty associated with the curvature of the film.

The difference between the predicted and measured doses was used to calculate the standard deviation, which was then considered as the fitting uncertainty. LAR was not applied to the films and this uncertainty was excluded from the uncertainty budget.

### 2.8 Curve fitting method

Guan et al. reported that the Radbard NIH (image) curve fitting method was superior to the typical rational function used in the FilmQA Pro™ software for EBT‐XD films ^18^. Accordingly, for this study we employed the Radbard NIH (image) curve fitting function, which is accessible using ImageJ software. Calibration curves passing through most dose points indicated a good fit. Similarly, R^2^ values were used to quantify the goodness of fit. The Radbard NIH (image) curve fitting is expressed by Equation (5):

(5) $$y\;=\;c*{\left(\frac{x-a}{d-x}\right)}^{\frac{1}{b}}$$

Here, a, b, c, and d are the fitting coefficients, and x represents the OD or PVs for a given dose. Solving for y allowed us to obtain the predicted dose using the curve‐fitting model. The curve‐fitting coefficients were determined for each calibration curve. The dose predicted by this model was evaluated against the known exposures of the films at doses of 0.5, 2, 5, 8, 12, 16, 20, 25, and 30 Gy, and the percentage difference between the predicted and measured doses was calculated. In addition, the first derivative of each color channel was computed to quantify the sensitivity of the film within a given dose range.

## 3 RESULTS

Table 1 lists the measurement uncertainties, with the blue channel exhibiting the largest uncertainty relative to the red and green channels. The fitting uncertainty was higher than those of the others.

**TABLE 1:** Uncertainty budge of film dosimetry in each color channel (%).

Uncertainty components	Red	Green	Blue
Output	0.72	0.72	0.72
Scanner uniformity	0.42	0.20	0.56
Positional uncertainty	0.10	0.10	0.10
Inter film uniformity	0.43	0.20	0.58
Fitting uncertainty	1.20	1.30	1.50
Total uncertainty(k = 1)	1.52	1.52	1.85
Expanded uncertainty (k = 2)	3.10	3.00	3.70

**FIGURE 4:**
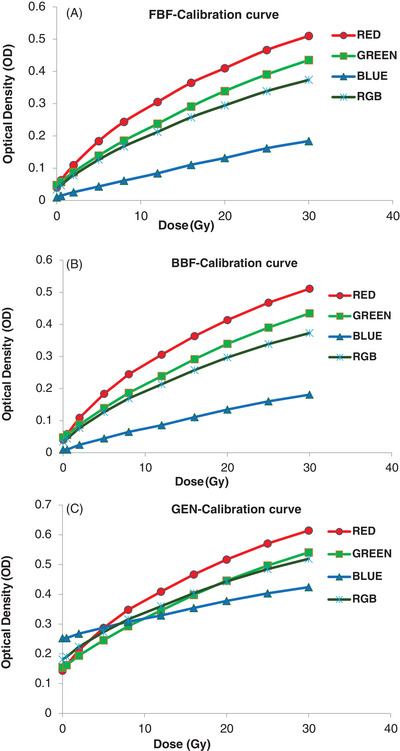
Calibration curves for the red, green, blue, and RGB channels obtained using the FBF, BBF, and GEN methods: (A) FBF method; (B) BBF method; (C) GEN method.

Figure 4 (A‐C) shows the calibration curves for the red, green, blue, and RGB channels obtained using the FBF, BBF, and GEN methods. The NIH fitting function was consistently applied to all calibration curves. Using the NIH model, the predicted dose was compared with the delivered dose in the range of 0.5 Gy to 30 Gy.

For the FBF calibration method, the average percentage difference for the red, green, blue, and RGB channels was 0.29%, 0.84%, ‐2.6%, and 0.37%, respectively. For the BBF calibration method, the average percentage differences between the predicted and delivered doses for the red, green, blue, and RGB channels were 1.35%, 1.9%, 6.1%, and 2.03%, respectively. Similarly, in the GEN calibration curve, the red, green, blue, and RGB channels exhibited differences of 1.4%, 1.9%, 6.1%, and 2.02%, respectively, between the predicted and delivered doses.

In the high dose range (5 Gy and above), the percentage differences between the predicted and delivered dose were as follows: For the FBF calibration method, for the red, blue, green, and RGB channels, they were ‐0.13%, ‐0.03%, 0.3%, and ‐0.05%, respectively. For the BBF method, for the red, green, blue, and RGB channels, they were ‐0.1%, ‐0.1%, ‐0.15%, and ‐0.15%, respectively. Finally, for the GEN method, for the red, green, blue, and RGB channels, they were ‐0.2%, ‐0.1%, ‐0.15%, and ‐0.15%, respectively.

**FIGURE 5:**
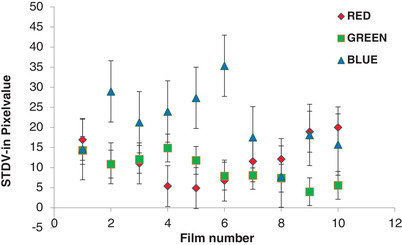
Standard deviation in the mean PVs of different films (inter‐film variation).

**FIGURE 6:**
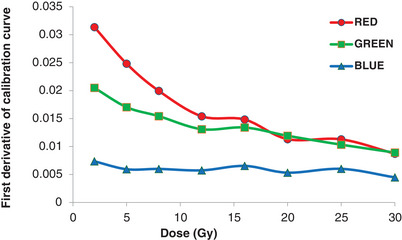
Sensitivity of EBT‐XD film in the red, green, and blue color channels.

Figure 5 illustrates the inter‐film variations in terms of the standard deviations of the PVs for the red, green, and blue channels. The largest variations in each channel were 30, 15, and 35, respectively. The sensitivity of the EBT‐XD film to each color is depicted as the first derivative in Figure 6. Both the red and green channels demonstrated superior sensitivity compared to the blue channel across the range of doses. The green channel surpassed the sensitivity of the red channel by approximately 16 to 20 Gy. The goodness of fit for each color channel was assessed using the R^2^ value (Table 2).

**TABLE 2:** R^2^ values of the fitting curves for the FBF, BBF and GEN methods.

FBF	R^2^	BBF	R^2^	GEN	R^2^
R	0.99992	R	0.99988	R	0.99988
G	0.99990	G	0.99988	G	0.99988
B	0.99953	B	0.99930	B	0.99930
RGB	0.99993	RGB	0.99982	RGB	0.99982

## 4 DISCUSSION

In this study, we conducted a quantitative analysis to assess the impact of different methods for selecting the unirradiated OD on the generation of calibration curves across different color channels. The OD variations among all the color and RGB channels were nearly consistent between the FBF and BBF methods. Statistical analysis indicated by *p*‐values demonstrated that the FBF and BBF methods did not significantly vary over the dose between 0.5 Gy and 30 Gy.

However, the OD changes in the GEN method differed significantly (*p* < 0.0001) from the FBF and BBF methods. In the FBF method, changes in the PVs of each calibrated film are accounted for by scanning before irradiation, which possibly eliminates inter‐film variations. In the BBF method, only one film from the same batch acted as a reference film, and its PVs were applied to all calibration films without considering inter‐film variations. The inter film uncertainties for the red, green, and blue channels were 0.43%, 0.2%, and 0.58%, respectively, which may impact the net OD results. This was not accounted for in the BBF scan mode, which could explain why the BBF method is slightly inferior to the FBF method for dose prediction.

In the GEN method, a common PV of 65535 was applied to all the calibration films, which also failed to account for inter‐film variations. The GEN method exhibited poor dose prediction, particularly in the blue channel, where significant fluctuations were observed in the region of interest. Santos et al. previously reported a 2.1% uncertainty in PVs for a 1 cm× 1 cm ROI in the blue channel, which aligns with our findings.^16^ In this study, the standard deviations of the blue channel were confirmed to be higher than those of the red and green channels. In terms of the fitting uncertainty, our study shows less deviation than other researchers,^20^ which may be due to the method of curve fitting used for the film analysis. In this study, the Radbard NIH (image) curve‐fitting method was used to accurately predict the dose. Guan et al. used the four‐parameter NIH Radbard function and showed that it generated a better fit than the three‐parameter rational function.^18^

Among the FBF, BBF, and GEN methods, FBF demonstrated promising results in predicting the dose in the red, green, and RGB channels, and its performance in the blue channel was acceptable for doses above 2 Gy. Perles et al. also used the FBF method calibration for high‐LET radiation and obtained satisfactory results.^21^ GEN, on the other hand, may introduce potential errors in dose prediction, particularly below 2 Gy where there can be up to a 6% difference. Our results are consistent with those of Miura et al., who reported that the blue channel exhibited a lower response gradient at any dose.^14^ The first‐derivative plots also confirmed that the blue channel was less sensitive than the other two channels. Although EBT‐XD films are specifically designed for high‐dose applications, their responses in the range of 2–5 Gy remain appreciable in the red, green, and RGB channels.

## 5 CONCLUSION

The choice of the method for obtaining the unirradiated optical density (background) can significantly affect the results of film dosimetry. For precise dose measurements using EBT‐XD films, it is advisable to use the FBF or BBF method rather than the GEN method, in which both inter‐and intra‐film variations are not considered. It is worth noting that the effects of different curve‐fitting methods on the FBF, BBF, and GEN methods were not investigated in this study, which can be considered one of its limitations. In addition, this study has the scope to verify gamma analysis for PSQA using different methods to choose the best calibration curve.

## CONFLICT OF INTEREST STATEMENT

The authors declare that they have no competing interests.

## ETHICS STATEMENT

Not applicable.
